# Letter to the Editor: AI and clinicians growing together: A cross-sectional survey of clinicians’ attitudes toward AI-CDSS with comparison to 2020 data

**DOI:** 10.1016/j.clinme.2026.100663

**Published:** 2026-10-29

**Authors:** Devon Ward, Ellesa Ahmad Nor Hisham

**Affiliations:** University of Liverpool, United Kingdom; University Hospitals of Liverpool Group, United Kingdom; University Hospitals of Liverpool Group, United Kingdom

Dear Editor,

We read Curiello *et al*.’s recent study which provides insight into contemporary clinician perspectives towards artificial intelligence-enabled clinical decision support systems (AI-CDSS) with interest.^1^ The authors’ results provide insight into contemporary attitudes of clinicians as well as outlining common concerns such as trainee de-skilling and loss of professional judgement. However, we think it is important to raise the question of whether the comparison with the Petkus *et al*. study can truly support conclusions regarding changes in clinician perspectives over time as suggested by the authors.^2^

Most substantially, the two studies sampled fundamentally different populations, a distinction which appears to be insufficiently characterised in the article. Curiello *et al*. sampled individual clinicians from the UK and Italy across multiple specialties.^1^ Petkus *et al*. sampled specialty society representatives and informatics leads, did not sample individual clinicians and did not report information on the respondents’ clinical status.^2^ We believe that comparing specialty society representatives and informatics leads with individual clinicians is methodologically problematic. A recent study found that familiarity with AI tools varies across different professional fields.^3^ This suggests that professional background may influence familiarity with and potentially attitudes towards AI. We argue this is important because the clinical status of Petkus *et al*.’s respondents was not reported, making it difficult to determine whether observed differences reflect changes in clinician perspectives over time, or differences in populations sampled.

Additionally, the authors deliberately expanded the sample to include respondents from Italy, although the rationale for doing so in the context of the UK-based Petkus *et al*. study is unclear. We argue that the addition of participants from different healthcare and geographical contexts is an additional source of heterogeneity in already unpaired and unmatched cohorts, complicating the temporal comparison with a UK-specific study.

Ultimately, while Curiello *et al*.’s study provides valuable contemporary data, the differences in sample populations and geographical contexts limit the comparative insight of clinicians’ perspectives that the authors seek to elucidate. Rather, using comparable populations or consistent longitudinal follow-up, can offer a more relative or temporal interpretation of clinicians’ attitudes towards AI-CDSS.

## CRediT authorship contribution statement

**Devon Ward:** Conceptualization, Writing – original draft, Writing – review & editing. **Ellesa Ahmad Nor Hisham:** Conceptualization, Writing – original draft, Writing – review & editing.

## Funding

This research did not receive any specific grant from funding agencies in the public, commercial, or not-for-profit sectors.

## Declaration of competing interest

The authors declare that they have no known competing financial interests or personal relationships that could have appeared to influence the work reported in this paper.
